# The feasibility of visualizing and quantifying muscle changes in postoperative oral cancer patients using Quantitative Muscle Ultrasound (QMUS)

**DOI:** 10.1007/s40477-024-00910-y

**Published:** 2024-06-19

**Authors:** Emily Vander Cruyssen, Jelmer van de Ven, Eric Dik, Simone Knuijt

**Affiliations:** 1 Department of Rehabilitation, Donders Institute for Brain, Cognition and Behaviour, Radboud University Medical Center, Nijmegen, The Netherlands; 2 Department of Rehabilitation, Regional Hospital Tienen (RZ Tienen), Tienen, Belgium; 3 Faculty of Medical Sciences, Radboud University, Nijmegen, The Netherlands; 4 Department of Oral and Maxillofacial Surgery, Radboud University Medical Center, Nijmegen, The Netherlands

**Keywords:** Quantitative muscle ultrasound, Orofacial muscles, Oral cancer, Speech therapy

## Abstract

**Purpose:**

Quantitative muscle ultrasound (QMUS) is a patient friendly tool for examining orofacial muscles. Resection of tissue can have an effect on the architecture and function of these muscles. The aim of this study is to investigate the feasibility of visualizing and quantifying muscle changes in postoperative oral cancer patients and to relate the findings to tumor and patient characteristics.

**Methods:**

Adult patients with a resected first primary pT1 or T2 oral squamous cell carcinoma, at least one year post operatively, where included. Ultrasound data were collected of the geniohyoid muscle, digastric muscles, masseter muscle, transverse muscle and genioglossus muscle. Ultrasound images were labeled as clearly visible, questionable or unclear. Of the clear muscles, echogenicity and muscle thickness were measured.

**Results:**

37 patients were included. The masseter muscle was clearly visible in all ultrasound images, both intrinsic tongue muscles had the lowest visibility (45.9%). There was a significant correlation between visibility and tumor localization for the genioglossus (*p* = 0.029). Age correlated with the visibility of the genioglossus muscle, BMI with the genioglossus and transverse muscles. Echogenicity and muscle thickness of the clearly identified muscles did not differ from normative values.

**Conclusion:**

QMUS of orofacial muscles is feasible in postoperative oral cancer patients with relatively small tumor sizes. Tongue resections negatively affected the visibility of the two intrinsic tongue muscles. These preliminary results for particular muscles indicate that the use of ultrasound might be promising in oral cancer patients to help determine targeted goals in post-operative rehabilitation.

## Introduction

Quantitative muscle ultrasound (QMUS) of orofacial muscles is a noninvasive, reproducible, reliable, safe and patient friendly tool for examining muscle structure and function [1–4]. QMUS is also time-efficient [5] and significantly less expensive than other imaging techniques [6]. Using QMUS, muscle thickness and echogenicity (EG) can be assessed [7]. EG can be quantified using histogram-based gray-scale analysis, which calculates the mean gray value of a region of interest. Based on normative values of muscle thickness and EG, z-scores can be calculated to compare QMUS data of affected and unaffected individuals [8]. An increased EG in muscles indicates unfavorable changes in the muscle architecture, which can lead to muscle dysfunction [9, 10].

Normal speech, swallowing and mastication relies on a complex composition of different types of orofacial muscles, each having a characteristic function. In oral tumor surgery, resection of tissue can have an effect on the architecture and function of orofacial muscles, because of their partial or complete removal. Ultrasound has been applied in oral cancer management in different phases of treatment [11, 12]. To our knowledge, it has not yet been used to analyze orofacial muscle quality in post operative oral cancer patients. The aim of this study is to investigate the feasibility of visualizing and quantifying muscle changes in postoperative oral cancer patients using QMUS and to relate the findings to tumor and patient characteristics. We hypothesized that resected muscles would be less visible through ultrasound measurements. Also, we hypothesized that there would be a significant correlation between muscle visibility and patient characteristics age, sex and BMI. Finally, we considered it possible to establish an increased muscle thickness in other orofacial muscles than the impaired, resected one because of compensatory behaviour.

## Materials and methods

**Fig. 1:**
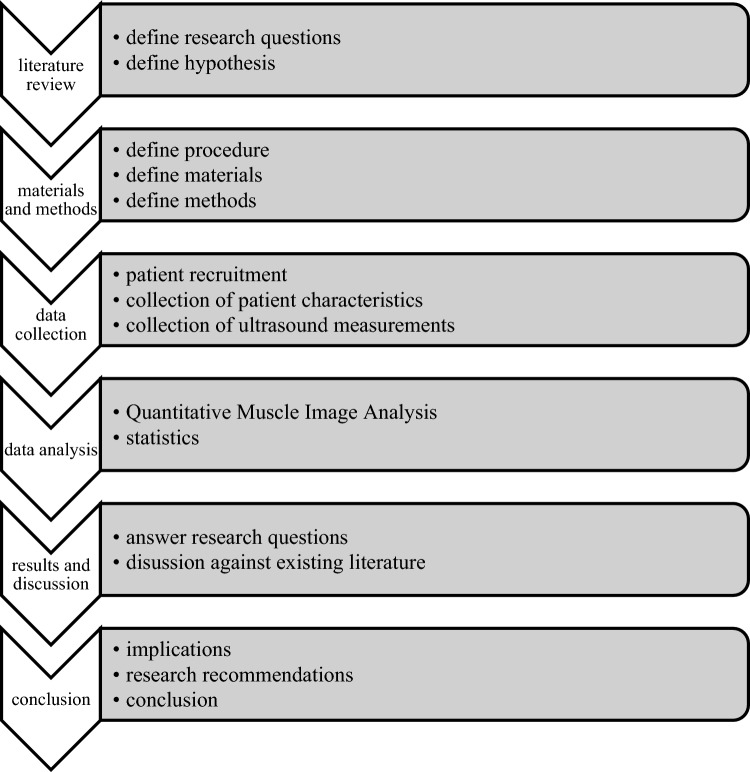
Diagram of the research process

See Fig. 1 for a diagram of the research process.

## Procedure

The surgical records of the department of maxillofacial surgery at the Radboud university medical center were searched for patients fitting the criteria. Suitable patients were called to ask if they wanted to receive an informational brochure about the study. Patients who agreed, were called seven days later to ask if they were interested in participating. An appointment was made for attending patients. All patients signed an informed consent. The medical ethic committee decided that this study did not require a Medical Research Involving Human Subjects Act certification, because orofacial muscle ultrasound is already part of usual care in the Radboudumc (file number 2022–13475).

### QMUS

**Fig. 2:**
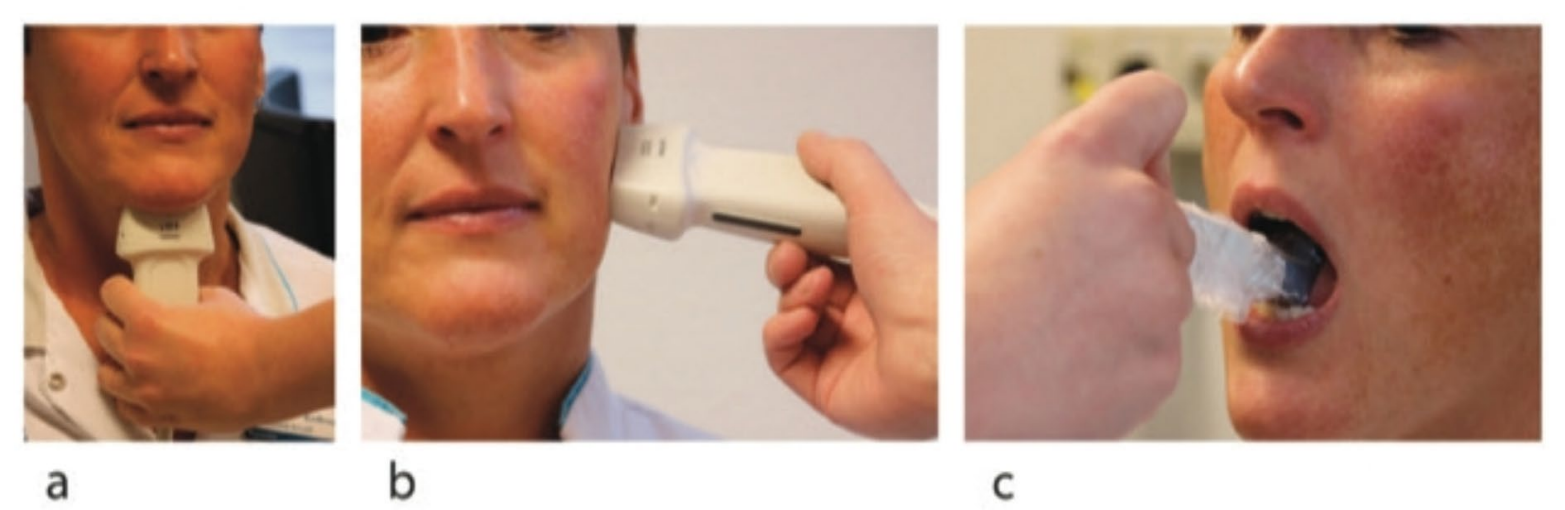
Positions of the ultrasound probes

The ultrasound data were collected using the Philips Affinity 70 (Philips Ultrasound, Inc.). Patients were sitting up straight on a chair and were asked to sit still and relax their orofacial muscles. Two types of probes were used: a regular linear probe and an intra-oral, so-called “hockey stick” probe (Fig. 2). The linear broadband probe 12–3 MHz was used for images of the floor of the mouth (a) containing the geniohyoid muscle and the digastric muscles and images taken from the side of the left jaw (b) containing the masseter muscle. The linear intra-oral “hockey stick” probe 15–7 MHz was used to image the tongue (c), containing the transverse muscle and genioglossus muscle. For this measurement, patients were asked to open their mouth and keep the tongue in a relaxed, low position. Each muscle was measured three times.

### Data-analysis

The ultrasound data were assessed using the QMUS software Qumia (Quantitative Muscle Image Analysis). First, Qumia was used to assess the visibility of each muscle. The ultrasound image was labeled ‘clear’ if the muscles were easily identified in all three ultrasound images, ‘questionable’ if doubts arose in at least one image, and ‘not visible’ if unclear in all three images.

Second, Qumia was used to select a region-of-interest in each ‘clear’ muscle to measure the EG. EG was expressed between 0 and 255 [13]. The presence of fibrosis in the muscle will cause a stronger reflection and thus a higher EG [14]. EG was converted into a z-score (the number of standard deviations from normal), which is calculated by Qumia based on normative data gathered in a large sample of healthy participants.

Third, muscle thickness of the masseter and the digastric muscles were measured using Qumia’s caliper tool in the clearest image. These scores were also converted into z-scores.

### Statistics

Patient characteristics are reported as the frequency and percentage for categorical variables; continuous variables are reported as the mean and standard deviation when normally distributed, or as the median and interquartile range (IQR) if not normally distributed.

Visibility for each muscle is conveyed through descriptive statistics, specified as a percentage (i.e. percentage clearly, not or questionable visible).

The impact of possible affecting factors on the visibility of each muscle was assessed by calculating the Pearson correlation coefficient for age and BMI and using the Chi-Squared test of independence for sex, tumor lateralization, tumor size and tumor localization.

Data were analyzed using IBM SPSS Statistics for Windows, version 27 (IBM Corp., Armonk, NY).

## Results

### Patients

Thirty-seven patients with a pT1T2 N0N1 M0 oral squamous cell carcinoma were included. Patient and tumor characteristics are depicted in Table 1.

**Table 1:** Patient and tumor characteristics

Mean age in years (SD)	62.7 (12.2)
Range	24-81
Sex n (%)	
Male	22 (60)
Female	15 (40)
Time Postoperative^a^ (years)	
Mean (SD)	3.0 (1.1)
Range	1.2 – 5.2
Mean BMI^b^ (SD)	26.4 (5.4)
Range	17.4 – 42.2
T-stage n (%)	
pT1	28 (75.7)
pT2	9 (24.3)
N-stage n (%)	
pN0	32 (86.5)
pN1	5 (13.5)
M-stage n (%)	
pM0	37 (100)
Tumor localization n (%)	
Tongue	26 (70.3)
Floor of the mouth	7 (18.9)
Processus alveolaris inferior	3 (8.1)
Processus alveolaris superior	1 (2.7)

^a^ Time form surgery till examination date

^b^ Body Mass Index on examination date

Tumors where mostly located at the tongue (70.3%). Closure of the resection area was most commonly done with primary closure (78.4%). In the other patients, healing was achieved by secondary intention.

### Muscle visibility and affecting tumor characteristics

Table 2 comprises the visibility of each muscle expressed in percentages.

**Table 2:** Muscle visualization

Muscle	Clear visibility *n* (%)	Questionable visibility *n* (%)	Not visible *n* (%)
Masseter	37 (100%)	0 (0%)	0 (0%)
Geniohyoid	36 (97.3%)	1 (2.7%)	0 (0%)
Digastric L	31 (83.8%)	4 (10.8%)	2 (5.4%)
Digastric R	30 (81.1%)	5 (13.5%)	2 (5.4%)
Genioglossus^a^	17 (45.9%)	15 (40.5%)	4 (10.8%)
Transverse^a^	17 (45.9%)	11 (29.7%)	8 (21.6%)

^a^One missing

The masseter muscle was clearly visible in all ultrasound images. The geniohyoid muscle in 97.3% and the left digastric muscle in 83.8%. Visualization of the left digastric muscle was not possible or questionable in three patients with a resection of the floor of the mouth (two times resection in the midline, one time left) and in three patients with a tongue resection (two times resection right, one time left). The visibility score of the right digastric muscle was 81.1%. Visualization of this muscle was not possible or questionable in two patients with a resection of the floor of the mouth (one time resection in the midline, one time left), four patients with tongue resection (all resections right) and one patient with resection of the right alveolar processus.

The visibility score of the genioglossus muscle and the transversus muscle was similar with clear visibility in 45.9% of the cases. Most patients (63.2% in the transversus muscle and 57.9% in the genioglossus muscle) had a tongue resection, 31.6% had a resection of the floor of the mouth. For the genioglossus, there was a significant correlation between visibility and tumor localization (*p* = 0.029). For the transverse muscle, there was a significant correlation between tumor lateralization and the visibility of this muscle with a chi-square statistic of 7.67 (*p* = 0.022). Tumor size was not significantly associated with the visibility of the oral muscles.

**Fig. 3:**
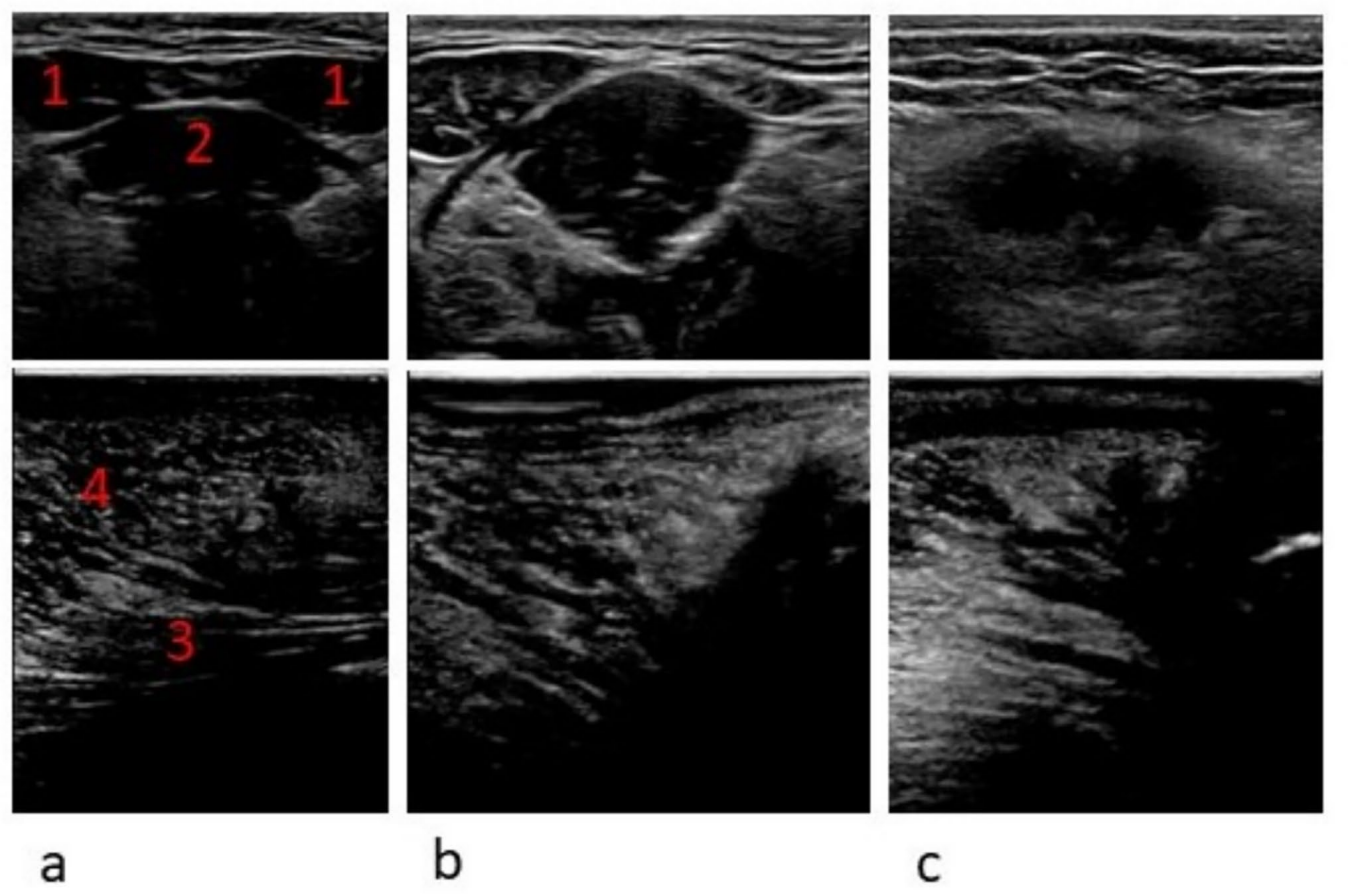
Clear (**a**), questionable (**b**) and unclear (**c**) ultrasound images of both digastric muscles (1), the geniohyoid muscle (2), the genioglossus muscle (3) and the transverse muscle (4)

Figure 3 shows clear, questionable and unclear ultrasound images of the orofacial muscles.

### Affecting patient characteristics

There is a low negative correlation between age and visibility of the genioglossus muscle (*p* = 0.030, *r* =− 0.362, *N* = 36), the visibility of the genioglossus muscle decreased as age increased. Other correlation coefficients concerning age and visibility were not significant. The correlation coefficients for BMI and visibility of the genioglossus muscle (*p* = 0.033, *r* = 0.357, *N* = 36) and BMI and visibility of the transverse muscle (*p* = 0.002, *r* = − 0.504, *N* = 36) are significant. There is a low to moderate negative correlation, meaning that visibility of the genioglossus muscle and transverse muscle decreased as the BMI increased. Other correlation coefficients concerning BMI and visibility were not significant.

Sex was not significantly associated with the visibility of the oral muscles.

### Echogenicity and muscle thickness

All clearly identified muscles were included in the calculation of EG (Table 3). A mean z-score of > 2 was perceived as deviant. There is no significant change in EG of the oral muscles compared to the normative data.

**Table 3:** Muscle echogenicity expressed in z-scores

Muscle	Clear visibility *n* (%)	Mean z-score (SD)	Range
Masseter	37 (100)	1.42 (1.7)	− 3.10 to 5.70
Geniohyoid	36 (97)	1.01 (1.6)	− 1.60 to 6.20
Digastric L	31 (84)	0.72 (2.8)	− 2.50 to 12.20
Digastric R	30 (81)	0.35 (1.8)	− 1.80 to 6.40
Genioglossus	17 (46)	0.76 (1.0)	− 0.90 to 2.40
Transverse	17 (46)	− 0.12 (1.3)	− 2.40 to 2.60

Muscle thickness of all clearly identified muscles was compared to muscle thickness found in a healthy population. A mean z-score of > 2 was perceived as deviant. Table 4 shows no significant changes in the muscle thickness of the oral muscles compared to normative values.

**Table 4:** Muscle thickness expressed in z-scores

Muscle	Clear visibility *n* (%)	Mean z-score (SD)	Range
Masseter	37 (100)	− 1.37 (2,7)	− 15.00 to 2.30
Digastric L	31 (97)	− 1.53 (2.0)	− 5.40 to 3.60
Digastric R	30 (81)	− 1.10 (1.7)	− 4.50 to 3.00

## Discussion

In this study we explored the use of quantitative muscle ultrasound to analyze orofacial muscles in post operative oral cancer patients. We found that the visibility of these muscles varied depending on the muscle we were looking at.

The masseter muscle was visible in each patient. This is obvious, because most patients had a resection of the tongue or the floor of the mouth, leaving the masseter muscle out of the area of surgery. The geniohyoid muscle also had a great, almost outstanding visibility. This is probably because we only included T1 and T2 tumors which need a relatively superficial resection. As the geniohyoid muscle is important for laryngeal elevation during swallowing [15], ultrasound might be feasible in T1 and T2 tumors among patients presenting with symptoms for diagnostic and therapeutic purposes. Future research can focus on the relationship between QMUS and patients with and without functional impairments.

Both digastric muscles were moderately visible. Ultrasound imaging of the two intrinsic tongue muscles resulted in low visibility scores. Patients with a tongue resection had a lower visibility of the genioglossus muscle. Thus, surgery of the tongue is associated with a decreased visibility in this muscle.

Reduced visibility might also result from a limited connection between the ultrasound probe and the tissue due to the presence of scar tissue. Scar tissue can cause an irregular surface which was actually the case in many of our patients [16]. This might interfere with an accurate seal, which can allow air to slip between the tongue surface and the probe. Air reflects 99% of the ultrasound wave [17], making it impossible to clearly visualize the muscles of the tongue. In addition, scar tissue can be seen as an artifact on ultrasound images with an appearance of an echogenic irregular area which may be surrounded by a hypoechoic halo [18].

Possible factors affecting the visibility of each muscle (i.e. age, BMI, tumor lateralization, sex) where explored. In our population, only the EG of the genioglossus muscle increased with age, which is in accordance with the literature [19]. However, it remains unclear why this association only applies on the genioglossus muscle. Furthermore, the visibility of the genioglossus muscle and transverse muscle decreased as BMI increased. These findings are in line with the literature, as the presence of subcutaneous fat can distort the ultrasound wave. This can cause the reflected echo to be refracted and attenuated [17]. Tumor lateralization was associated with visibility of the transverse muscle. The transverse muscle runs across the entire surface of the tongue. Therefore, we assessed whether this association resulted from tumor size, because larger tumors at one side of the tongue might impact visualization more than smaller ones. However, there was no association between tumor size and visualization for the transverse muscle nor any other orofacial muscle. This finding might result from the selection of the groups, as the sample includes only patients with smaller carcinomas (T1 and T2). Tumor localization was only associated with the visibility of the genioglossus. The lacking association between tumor localization and muscle visibility of other muscles is rather surprising, looking at the frequencies. For example, in patients with a resection of the floor of the mouth, visualization of the digastric muscles was not possible or questionable in three out of seven patients for the left digastric muscle and two out of seven patients for the right digastric muscle. This tendency needs further investigation in a larger sample.

There was no impact found of sex. We would have expected a possible association between sex and visualization of orofacial muscles, as muscles in woman tend to be easier to visualize through ultrasound than men [17]. This effect might be found when examining a larger sample.

There were no significant changes found in the EG nor muscle thickness of the clearly identified orofacial muscles compared to normative values. Normal muscle tissue has a black appearance because there are few tissue transitions between muscle fibers themselves and their surrounding connective tissue layers, blood vessels and nerves [3]. Stronger reflections represent more echogenic tissue. An increased EG of the orofacial muscles would thus show an altered muscle histology, such as the presence of fat. We conclude that muscle histology remained unchanged in all clearly identified orofacial muscles. The unchanged muscle thickness in orofacial cancer patients indicates no compensatory muscle use post-surgery. We considered it possible to establish an increased muscle thickness in other orofacial muscles than the impaired, resected one. In this case, the remaining muscle(s) would compensate for the decreased activity in the impaired muscle. A comparison with existing evidence is not possible, since our study is the first to look into muscle composition in oral cancer patients through ultrasound.

## Limitations

This study has some limitations. We only included patients with a relatively small tumor size who had surgery with mainly primary closure. Hence, there was no opportunity to look into the effect of tumors closed with a free flap reconstruction. Several gaps remain in interpreting the current results, such as the lack of a consistent association between different factors like tumor size, tumor localization and tumor lateralization. These gaps need further investigation in a wider sample of patients, where each group of patients has a large sample size. Furthermore, functional impact was not part of the selection of participants. Patients with functional impairments, such as speech or swallowing problems, might show different imaging results through ultrasound. This could be the subject of future research.

## Conclusion

QMUS of orofacial muscles is feasible in postoperative oral cancer patients with relatively small tumor sizes. Tongue resections negatively affected the visibility of the two intrinsic tongue muscles. These preliminary results for particular muscles indicate that the use of ultrasound might be promising in oral cancer patients to help determine targeted goals in post-operative rehabilitation.

## Data Availability

The data that support the findings of this study are available on reasonable request from the corresponding author (SK).
